# Potential Neuroprotective Mechanisms of Methamphetamine Treatment in Traumatic Brain Injury Defined by Large-Scale IonStar-Based Quantitative Proteomics

**DOI:** 10.3390/ijms22052246

**Published:** 2021-02-24

**Authors:** Shichen Shen, Ming Zhang, Min Ma, Sailee Rasam, David Poulsen, Jun Qu

**Affiliations:** 1 Department of Pharmaceutical Sciences, University at Buffalo, Buffalo, NY 14214, USA; ShichenS@buffalo.edu (S.S.); MZhang25@buffalo.edu (M.Z.); 2 New York State Center of Excellence in Bioinformatics and Life Sciences, Buffalo, NY 14203, USA; MinMa@buffalo.edu (M.M.); SaileeSu@buffalo.edu (S.R.); 3 Department of Cell Stress Biology, Roswell Park Comprehensive Cancer Institute, Buffalo, NY 14203, USA; 4 Department of Biochemistry, Jacobs School of Medicine and Biomedical Sciences, University at Buffalo, Buffalo, NY 14203, USA; 5 Department of Neurosurgery, Jacobs School of Medicine and Biomedical Sciences, University at Buffalo, Buffalo, NY 14203, USA

**Keywords:** traumatic brain injury, methamphetamine, large-scale proteomics, mass spectrometry, IonStar

## Abstract

Although traumatic brain injury (TBI) causes hospitalizations and mortality worldwide, there are no approved neuroprotective treatments, partly due to a poor understanding of the molecular mechanisms underlying TBI neuropathology and neuroprotection. We previously reported that the administration of low-dose methamphetamine (MA) induced significant functional/cognitive improvements following severe TBI in rats. We further demonstrated that MA mediates neuroprotection in part, via dopamine-dependent activation of the PI3K-AKT pathway. Here, we further investigated the proteomic changes within the rat cortex and hippocampus following mild TBI (TM), severe TBI (TS), or severe TBI plus MA treatment (TSm) compared to sham operated controls. We identified 402 and 801 altered proteins (APs) with high confidence in cortical and hippocampal tissues, respectively. The overall profile of APs observed in TSm rats more closely resembled those seen in TM rather than TS rats. Pathway analysis suggested beneficial roles for acute signaling through IL-6, TGFβ, and IL-1β. Moreover, changes in fibrinogen levels observed in TSm rats suggested a potential role for these proteins in reducing/preventing TBI-induced coagulopathies. These data facilitate further investigations to identify specific pathways and proteins that may serve as key targets for the development of neuroprotective therapies.

## 1. Introduction

Traumatic brain injury (TBI) accounts for over 2.8 million emergency department visits and over 56,000 deaths in the United States annually [1]. TBI involves primary structural injury triggered by external force on the brain followed by secondary injury events occurring over prolonged timelines. The combination of primary and secondary injuries activates a cascade of neuropathological signaling pathways that involve neuroinflammation, reactive oxygen species (ROS) production, calcium dysregulation, blood–brain barrier disruption, excitotoxicity, necrotic cell death, autophagy, and apoptosis [2]. Because of the highly complex and heterogeneous nature of TBI [3], a comprehensive and in-depth systems biology approach is warranted to understand the molecular mechanisms underlying TBI-induced neuropathy and to better facilitate the clinical management of TBI patients and the development of promising therapeutic drug candidates. However, this remains challenging due to the incapability of most experimental techniques to simultaneously target hundreds or thousands of molecules in one single experiment.

Mass spectrometry-based quantitative proteomics represents a promising experimental approach that supports unbiased investigation of protein abundance changes from various biological samples [4]. Such a system biology-based approach can be applied to large sample cohorts, rendering it a powerful tool to define critical biological processes and signaling pathways dysregulated after TBI insults and discovering putative circulatory or tissue-specific biomarkers for TBI diagnosis and prognosis. In the past decade, a number of studies have applied quantitative proteomics to interrogate the neuroproteomic profiles of brain tissues procured from clinical patients or animal models of TBI [5,6,7,8,9]. While results reported vary considerably owing to the differences in proteomic methods applied, sample origin, TBI models, and time frames, these studies have started to address the gaps in our current knowledge regarding the signaling transduction networks involved in TBI-induced neuropathy.

In the current study, we adopted a peptide-centric, discovery-driven quantitative proteomics approach to investigate proteomic changes within the rat cortex and hippocampus at 32 h post TBI. We specifically compared changes between mild and severe TBI induced by the lateral fluid percussion injury (FPI) method. We previously demonstrated the neuroprotective potential of low-dose methamphetamine (MA) following severe TBI in the rat lateral FPI injury model [10,11,12,13]. In an effort to further delineate the putative mechanisms underlying the neuroprotective effects of low-dose MA, we investigated the unique proteomic changes induced by MA administration following severe TBI compared to both mild and severe TBI. IonStar, a unique label-free MS1 ion-current-based quantitative proteomics pipeline, tailored to resolve the technical hurdles in large-scale quantitative proteomics, was applied in this study [14]. IonStar allows in-depth and reliable proteomics characterization in large biological sample cohorts with high accuracy/precision, and an extremely low missing data and false biomarker discovery rate, pertinent to the purpose of the current study [15]. Quantitative results were analyzed by multiple informatics approaches to identify potential proteins and corresponding signaling pathways that were significantly altered within each treatment group that could serve as potential targets for neurotherapeutic development.

## 2. Results

### 2.1. In-Depth Characterization of Rat Brain Proteome Using IonStar

**Figure 1:**
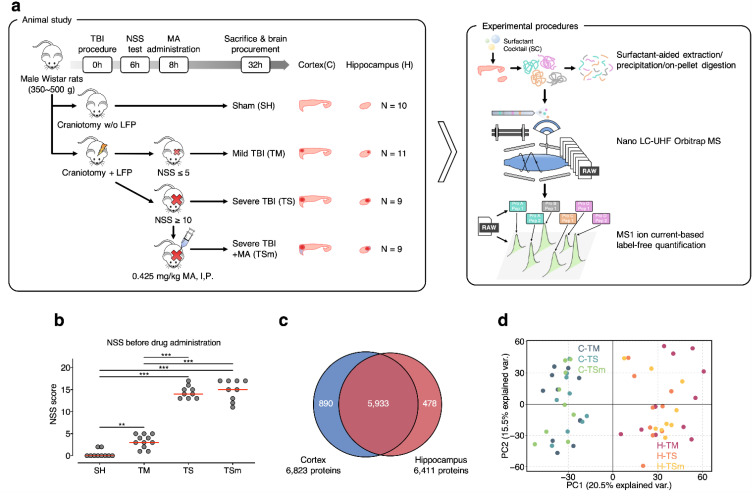
Quantitative proteomics profiling of cortical and hippocampal tissues procured from a rat lateral fluid percussion injury (FPI) model of traumatic brain injury (TBI). (**a**) The scheme of animal study and proteomic experimental procedures; (**b**) Neurological Severity Score (NSS) test results of animals involved in the study. Double asterisks (**) and triple asterisks (***) denote *p* < 0.01 and *p* < 0.001 determined by one-way ANOVA + Tukey honestly significant difference (HSD) test; (**c**) Venn diagram of proteins quantified in cortex and hippocampus samples; (**d**) principal component analysis (PCA) of 5933 proteins quantified in both the cortex and hippocampus.

In the current study, a large-scale neuroproteomics investigation of the clinically-relevant rat lateral FPI model of TBI was conducted. The scheme of animal study and experimental procedures are illustrated in Figure 1a. The lateral FPI model was selected to recapitulate the neuropathological features of closed head TBI. Injury severity was defined based on functional outcomes assessed by the Neurological Severity Score (NSS). The group average NSS at 6 h after TBI for sham (SH), mild TBI (TM), severe TBI (TS), and severe TBI + methamphetamine (TSm) rats in this study were 0.4 ± 0.8, 3.2 ± 1.5, and 14.4 ± 1.4 and 14.9 ± 2.4, respectively, as shown in Figure 1b. These scores fell within the defined ranges for mild (0–5) and severe (11–18) TBI and demonstrate that all severe TBI rats received a similar injury severity. IV infusion of saline or methamphetamine was initiated at 8 h post TBI and continued continuously for 24 h, consistent with our previously reported dosing regimen. Brain tissue was collected at the end of the 24-h infusion, which was 32 h after TBI. The cortex and hippocampus were separated from the whole brain and analyzed.

To achieve in-depth and reliable quantification of the rat cortex and hippocampus proteome, we adopted an integrated label-free quantitative proteomics approach, IonStar [14]. IonStar consists of four major components: (i) a surfactant cocktail-aided extraction/precipitation on-pellet digestion (SEPOD) sample preparation protocol to achieve exhaustive and efficient yields of tryptic peptides from brain samples rich in membrane-bound proteins; (ii) a nano liquid chromatography-mass spectrometry (LC-MS) procedure on a high-resolution Q/Ultra-high-field (UHF) Orbitrap mass spectrometer with an optimized trapping/delivery strategy to provide ultra-high sensitivity and reproducibility for peptide detection; (iii) an MS1 ion-current-based quantitative method to enable confident and robust generation of quantitative features; (iv) an experimental null-based approach to allow experimental determination of the optimal cutoffs for significantly altered proteins (APs) [16]. The SEPOD protocol provided highly efficient and reproducible protein (86.5 ± 7.2 μg/mg tissue) and peptide (~90%) yields from the 39 cortex and 39 hippocampus samples with excellent recovery for membrane-bound proteins (~26.9% protein IDs identified were enriched in membrane-related Gene Ontology (GO) terms). The optimized trapping-nano LC-Orbitrap MS system coupled with a unique MS1 ion-current-based quantitative approach enabled the quantification of 6823 and 6411 unique protein groups (7301 in total) in the cortex and hippocampus, respectively, with <0.5% missing data rate on the protein level, as shown in Figure 1c. Intra-group coefficient of variation (CV) was 16.7% and 5.0% on the protein level for biological and technical replicates, as shown in Figure S1, implicating excellent quantitative precision by IonStar. Moreover, proteins quantified spanned ~6 orders of magnitude in terms of ion MS1 intensities, as shown in Figure S2, covering both high-abundance housekeeping proteins as well as low-abundance regulatory proteins involved in various signaling pathways. According to our knowledge, this is by far the first TBI proteomics study achieving a quantitative depth of >7000 unique protein groups and excellent quantitative quality in a large-cohort sample set. Protein quantification results are enclosed in Tables S1 and S2.

As demonstrated by our previous studies, MA administration with a lowest effective dose of 0.25 mg/kg/h was capable of mitigating neurological dysfunction and cognitive impairment in the rat lateral FPI model of TBI when administered 12 h post severe TBI [11]. We previously reported that MA acted via a dopamine-PI3K-AKT signaling cascade that resulted in the suppression of apoptotic neuronal death, and increased neurogenesis within the dentate gyrus [12]. In addition, we reported gene expression data that suggests the neuroprotective effects of low-dose MA may also be related to decreased expression of proinflammatory genes including CCL2, MyD88, and IL1β [13]. In this current study, we aimed to explore how the same MA treatment would alter the global protein expression profiles within the rat cortex and hippocampus following severe TBI. Principal component analysis (PCA) of protein ratios between each TBI group and SH, as shown in Figure 1d, was performed with the 5933 protein groups quantified in both the cortex and hippocampus. Clear segregation between cortex and hippocampus samples was observed, suggesting disparities between proteomic patterns in the two brain regions. Interestingly, we found that TM and TS samples were more separated from each other. In contrast, proteomic patterns observed after severe TBI and MA treatment (i.e., TSm) were more similar to TM. This was confirmed by Pearson correlation of the same data, as shown in Figure S3, where higher R-squared values between TSm and TM were observed compared to those seen between TS and TM, suggesting that MA treatment induced proteomic patterns that more resembled mild injury rather than severe. In addition, the R-squared values between the cortex and hippocampus was extremely low, which also corresponds well with PCA results.

### 2.2. Comparison of Global Protein Changes Post TBI and MA Treatment 

**Figure 2:**
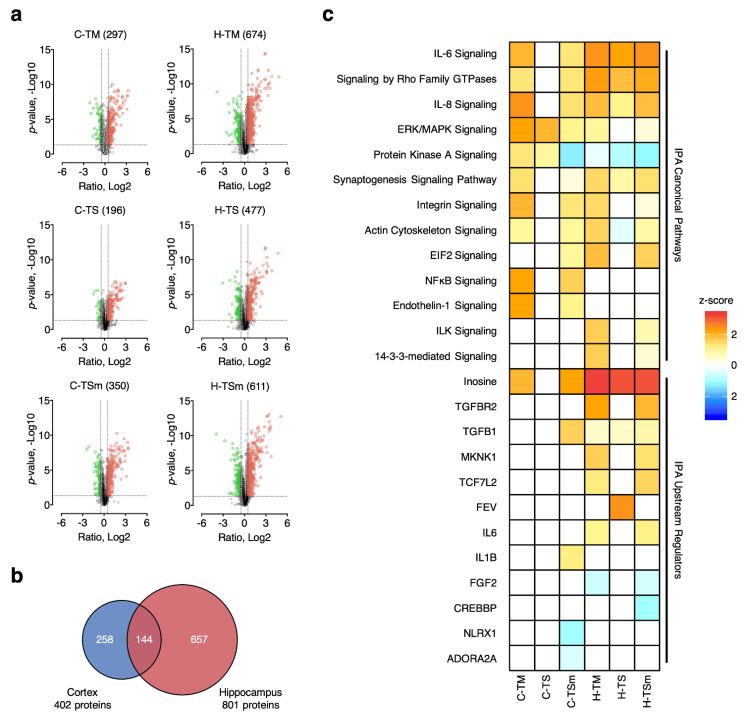
Determination of altered proteins (APs) in mild TBI (TM), severe TBI (TS), and severe TBI + methamphetamine (MA) (TSm) groups. (**a**) Volcano plots of protein ratios (x-axis) and *p*-values (y-axis) for each TBI group; (**b**) Venn diagram of APs identified in the cortex and hippocampus; (**c**) canonical pathways (upper) and upstream regulators (lower) enriched from APs by Ingenuity Pathway Analysis (IPA).

Based on the results above, we hypothesized that on the proteome level, the effects of MA on severe TBI could possibly be represented by two general types of protein changes. One type of change observed following MA treatment represents the absence of downstream aberrant protein level changes observed in severe TBI conditions, thus leading to a pattern more similar to mild TBI. These types of mechanisms would reflect an “effectual” change that occur as a consequence of upstream events blocking neuropathology. These “effectual” protein changes are classified as MA-induced changes in the current study. Alternatively, MA could uniquely and specifically activate certain critical neuroprotective signaling pathways that alleviate neuronal death and neuropathology. These types of mechanisms would reflect a “causal” change and are classified as MA-unique changes in the current study. We hypothesized that these MA-induced “causal” changes possibly encompass pathways that were critical and specific for the neuroprotective effects of low-dose MA. Therefore, in an effort to classify protein changes associated with MA treatment as “effectual” or “causal”, we first defined the proteins that were significantly altered in each group. Most commonly, altered proteins (APs) are determined by statistical testing (e.g., Student’s t-test, one-way ANOVA) with optional adjustment by multiple testing correction. However, it has been reported that multiple testing correction may not be necessarily optimal for proteomics data due to several factors, limiting its application [17]. Alternatively, protein fold change (FC) has been introduced as an additional criterion. Nonetheless, currently the selection of protein FC cutoff is largely arbitrary or experience-based, which may be an issue for proteomic sample sets with less prominent change levels or higher biological variation. To address this issue, we developed an experimental null (EN) approach, which establishes the null distribution of the sample set using the control group and evaluates the false discovery rate under a series of common FC thresholds [16]. The false altered-protein discovery rate (FADR), defined as the ratio of the number of significantly altered proteins between case-control comparison and EN, was calculated under multiple protein FC thresholds (i.e., 1.2, 1.3, 1.4, 1.5, and 2) with a *p*-value of <0.05, and plotted for all sample groups involved, as shown in Figure S4. Taking the MA-treated group (i.e., TSm) as an example, significant decrease of FADR is observed when the cutoff for protein FC elevates from 1.2 to 1.4 (11.3% to 4.0% and 6.2% to 2.3% for cortex and hippocampus), while raising the protein-fold-change cutoff to 1.5 does not result in much decrease of FADR (<0.5%). Moreover, although using 2-fold protein change as the cutoff allows further decrease of FADR (1.0% and 0.6% for cortex and hippocampus), the number of APs also declines by 65.0% and 72.5% compared with 1.4-fold cutoff. Based on the consideration to simultaneously control FADR at a low level and ensure a reasonable number of APs, we selected a 1.4-fold protein change as the final cutoff thresholds, along with the *p*-value cutoff of <0.05. As a consequence, 402 and 801 APs were identified in at least one of the treatment groups in the cortex and hippocampus, respectively, as shown in Tables S3 and S4. Surprisingly, more APs were identified in the TM and TSm groups as opposed to TS rats, as shown by Volcano plots in Figure 2a. On the other hand, proteins that significantly changed in the cortex and hippocampus appear to be very different from each other, with a low overlapping rate of 31.5%, as shown in Figure 2b. This suggests that TBI and MA treatment induced highly divergent protein changes in the cortex and hippocampus at 32 h after injury. High similarity of protein changes between TSm and TM was also observed (77.9% for cortex, 79.5% for hippocampus), substantiating our hypothesis that one of the potential mechanisms of MA-induced neuroprotective effects is the restoration of aberrant protein expression levels in an irreversible severely injured brain to a pattern more resembling the recoverable, mildly injured brain.

In order to acquire better understanding of which biological functions and signaling pathways are regulated by MA treatment, Gene Ontology (GO) annotation and pathway analysis was performed. GO Biological Processes (GOBP) from the Database for Annotation, Visualization, and Integrated Discovery (DAVID) as well as Canonical Pathways/Upstream Regulators from Ingenuity Pathway Analysis (IPA) enriched are presented in Figure S5 and Figure 2c. The GOBP terms associated with the APs include three major functional categories related to: (1) neuroinflammation, including cellular response to IL-6, complement activation classical pathway, innate immune response, and acute-phase response; (2) blood circulation, including oxygen transport, vasodilation, fibrinolysis, and platelet aggregation; (3) cell survival and proliferation, including astrocyte development, negative regulation of apoptotic process, cell adhesion, and positive regulation of cell death. Signaling pathways with reported roles in TBI and other neurological diseases were also enriched, including IL-6/IL8 signaling, ERK/MAPK signaling, Rho GTPase signaling, integrin signaling, RhoGDI signaling, and NFKB signaling. PKA signaling and actin cytoskeleton signaling, more related to the psychostimulating effects of MA, were also enriched. Notably, the pattern of enriched GO terms and canonical pathways appears to be more similar between TM and TSm groups, which suggests that biological processes and signaling pathways activated in TM conditions were also activated with MA treatment in TS conditions. This again supports the proposed restoration mechanism by MA treatment in a severely injured brain.

Moreover, analysis of the APs revealed an interesting pattern of predicted changes associated with the activation of upstream regulatory proteins. IPA again identified similar patterns of changes within the TM and TSm groups that were distinct from the TS groups. The majority of changes to regulator activity was observed in hippocampal tissue. Specifically, activation of signaling through TGFβR2, MKNK1, TCF7L2, and IL-6 were predicted in hippocampal tissue from TM and TSm rats compared to TS rats, which showed no change in the status of these regulators, as shown in Figure 2c. Interestingly, while a moderate increase in TGFβ1-mediated signaling was seen in hippocampal tissue from all groups, a substantial increase was observed only in cortical tissue collected from TSm rats.

### 2.3. Classification of APs into MA-Induced and MA-Unique Subgroups 

**Figure 3:**
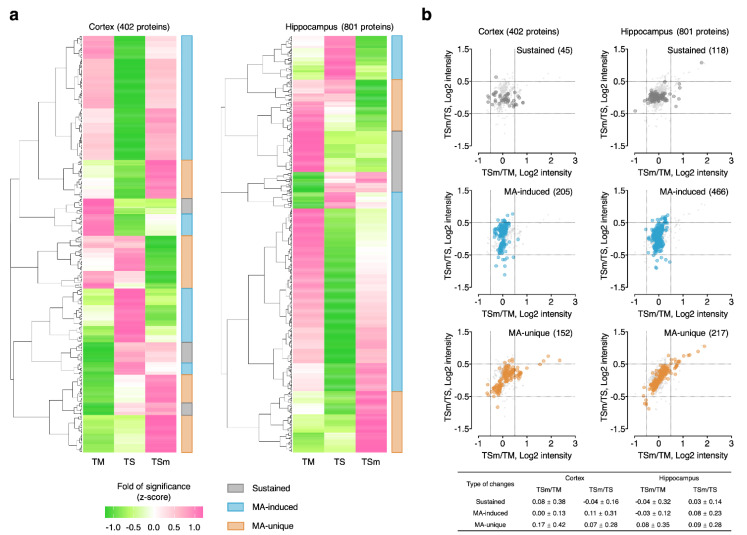
Classification of APs into different groups with hierarchical clustering. (**a**) Heatmaps of hierarchical clustering results. APs were classified into sustained APs, MA-induced APs, and MA-unique APs based on fold of significance, a combined score of protein fold change and *p*-value; (**b**) validation of AP classification results by correlation of TSm/TM and TSm/TS ratios.

To further define biological functions and signaling pathways specifically regulated by low-dose MA administration, hierarchical clustering of the APs in the cortex and hippocampus was performed using a normalized fold of significance (i.e., z-score) integrating both protein FC and *p*-value, as shown in Figure 3a. Consequently, APs were classified into three categories: (1) sustained APs, which showed consistent z-scores with and without MA treatment and were consistently associated with severe TBI; (2) MA-induced APs, which showed similar z-scores between TM and TSm groups, suggesting that the protein changes dysregulated in severe TBI were normalized to a level similar to mild TBI with MA treatment and represent “effectual” changes; (3) MA-unique APs, which showed distinct z-scores in TSm rats, suggesting that the protein changes observed were uniquely associated with MA treatment and represent “causal” changes. Hierarchical clustering analysis resulted in 45/118 sustained APs, 205/466 MA-induced APs, and 152/217 MA-unique APs in the cortex and hippocampus, respectively, as shown in Tables S3 and S4. To confirm whether clustering of the APs by the normalized fold and significance gave valid results, we retrospectively compared scatterplots for sustained, MA-induced, and MA-unique APs using TSm/TM ratio as the x axis and TSm/TS as the y axis. We anticipated that sustained APs would exhibit higher FC in the TSm/TM dimension since MA treatment did not induce changes in these proteins relative to TS. In contrast, we anticipated that MA-induced APs would exhibit higher FC in the TSm/TS dimension since MA treatment restored protein levels more in line with TM. Finally, we anticipated that MA-unique APs would exhibit a more random distribution since MA treatment induced changes specific to the TSm group. Accordingly, we observed these corresponding patterns for the three classes of protein changes in both the cortex and hippocampus, as shown in Figure 3b, which indicates the classification procedure by hierarchical clustering faithfully reflects the designated patterns of protein changes. 

**Figure 4:**
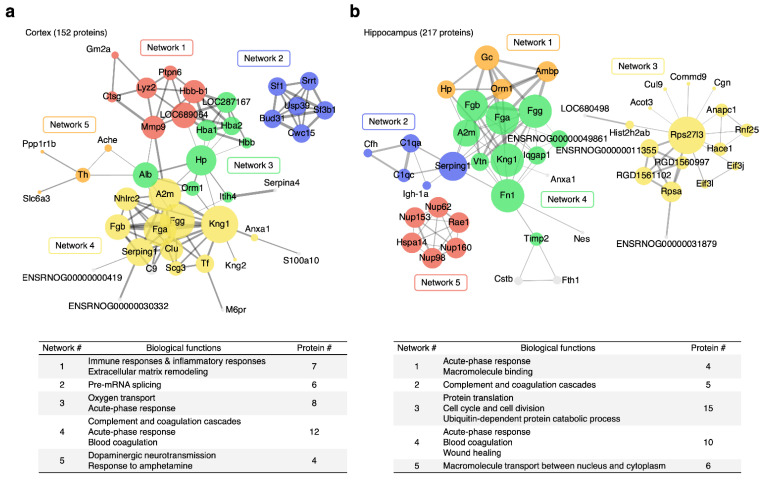
Protein–protein interaction (PPI) networks enriched from MA-unique APs in (**a**) cortex and (**b**) hippocampus using STRINGDB. Only networks with high confidence (STRING score > 0.7) and >3 nodes were retained.

The MA-unique APs were further dissected into functional networks relevant to the neuroprotective effects of MA using STRINGDB. As shown in Figure 4, a number of protein–protein interaction (PPI) networks with high confidence (STRING score > 0.7) and >3 nodes were successfully identified in both brain regions. By referencing nodes back to results of the functional analysis above, we were able to further elucidate the biological relevance of each PPI network and further define the potential mechanisms underlying the neuroprotective effects of MA. In the cortex, PPI networks were mainly involved in immune and inflammatory responses, pre-mRNA splicing, acute-phase response, oxygen transport, complement and coagulation cascades, and dopaminergic neurotransmission. In the hippocampus, PPI networks were mainly involved in acute-phase response, complement and coagulation cascades, protein translation, cell cycle and cell division, blood coagulation/wound healing, and macromolecule transport between nucleus and cytoplasm. 

**Figure 5:**
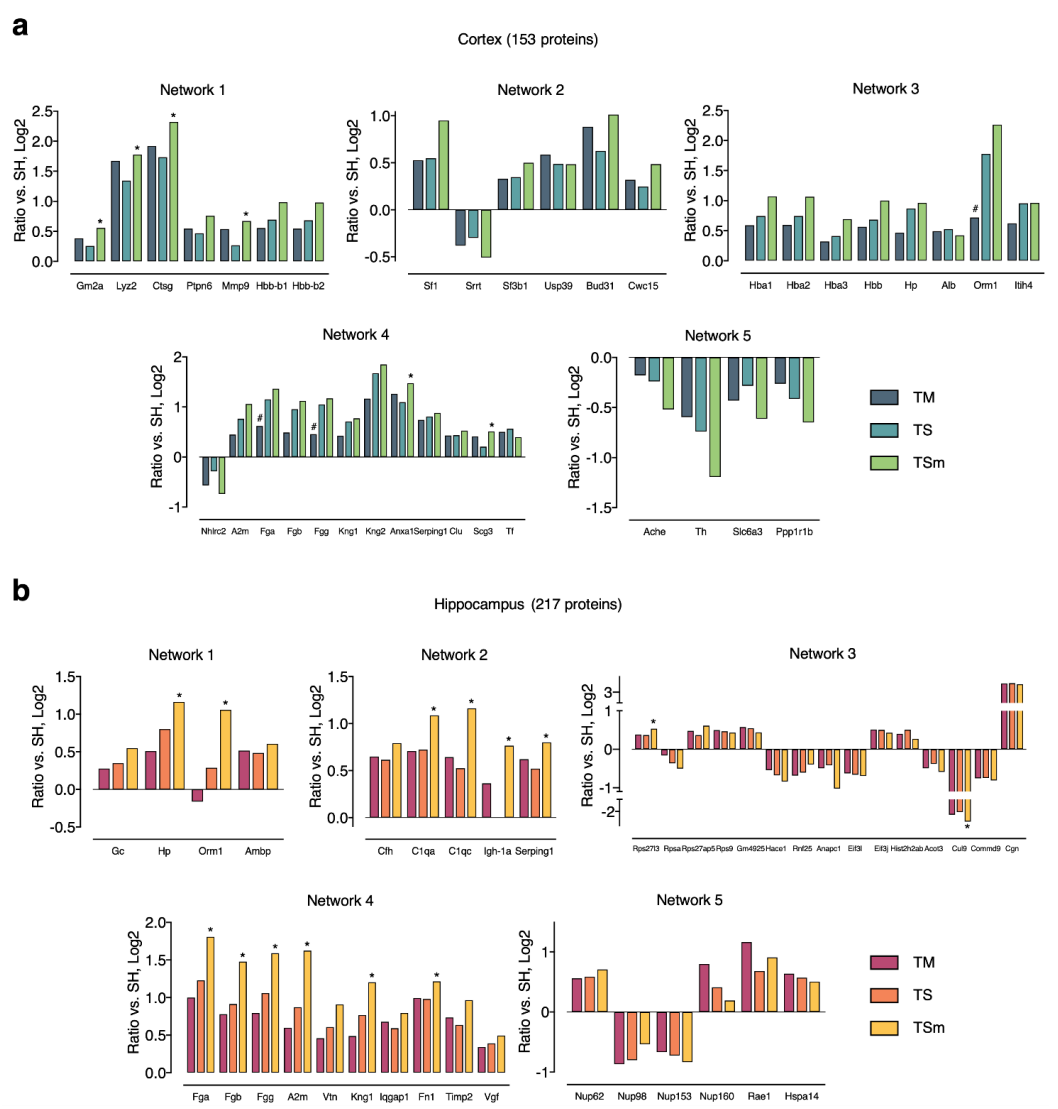
Expression profiles of MA-unique APs from each PPI network in (**a**) cortex and (**b**) hippocampus. Asterisks indicate statistical significance (i.e., *p* < 0.05) between TS and TSm, while hash signs indicate statistical significance (i.e., *p* < 0.05) between TS and TM.

Figure 5 shows protein ratios of all nodes involved in the TSm-enriched PPIs for all treatment groups (TM, TS, and TSm). The greatest number of APs within the cortex were seen in Network 1, with 57% of proteins (4/7) observed within this network being significantly different between TS and TSm rats. Network 1 involves immune and inflammatory responses as well as extracellular matrix remodeling. The greatest number of significantly altered proteins overall between TS and TSm rats were observed within the hippocampus, with 80% of proteins within Network 2 and 60% of the proteins observed in Network 4, being significantly different between TS and TSm rats. Hippocampal Network 2 involves the complement and coagulation cascade, while Network 4 involves acute phase response, blood coagulation, and wound healing. 

## 3. Discussion

### 3.1. Global Patterns of Alterations in Neuroproteomics

In the current study, we adopted IonStar, a reliable MS1 ion-current-based quantitative proteomics approach for large-cohort proteomics analysis, in combination with an experimental null-based method that enables empirical determination of the optimal cutoffs for AP discovery, to obtain in-depth quantification of the rat cortex and hippocampus neuroproteome following either mild or severe TBI. We also included low-dose MA treatment as a tool to probe the potential molecular mechanisms involved in neuroprotection elicited by this drug when administered following severe TBI. Unexpectedly, we observed more APs in TM and TSm than in TS rats. In addition, we observed greater changes within the hippocampus compared to the cortex at the 32-h time point measured here, which is consistent with our previous observations that histological changes were more prominent within the hippocampus at acute time points (48 h) following severe TBI [13,18]. 

We previously published data on the levels of specific miRNAs within the plasma collected from the same rats used in the present study. We reported that the levels of miR-183, miR-200a-3p, miR-200b-3p, and miR-429 detected by RNAseq analysis and validated by ddPCR within the plasma of TSm rats at 32 h post severe TBI were significantly lower than the levels we detected in TS [19]. In contrast, the levels of these specific miRNAs in TSm rats were not significantly different from those seen in TM rats. Consistent with this observation, we observed here that the neuroproteome profile of TSm rats was also consistently similar to that seen with TM rather than TS rats. The proteomics data we present here further confirms that acute treatment of severe TBI rats with low dose methamphetamine produces both miRNA and proteomics profiles that are more similar to mild TBI rather than severe TBI. These data are also consistent with our previously published observations that acute treatment with low dose methamphetamine results in improved functional and cognitive improvements following severe TBI in the fluid percussion injury model. 

### 3.2. Alteration in Canonical Pathways 

This general pattern of similar neuroproteome profiles observed between TM and TSm rats was further reflected in IPA canonical pathways. Interestingly, our data indicated that the levels of proteins associated with the IL-6 signaling pathway were increased in the hippocampus for all three groups. In contrast, increases were observed only in the cortex of both TM and TSm rats but remained unchanged for TS controls. Although IL-6 signaling activity was increased in TM and TSm rats, we did not detect a corresponding increase in IL-6 levels in these rats. While chronic elevation of IL-6 can result in a deleterious proinflammatory response, acute and short-term activation of IL-6 signaling in the TM and TSm rats may represent a potential neuroprotective mechanism in TBI. This hypothesis is supported by the observation that IL-6 mediates neuroprotection against NMDA-induced excitotoxicity in part through PI3/AKT signaling [20,21,22]. In relation to this observation, we have demonstrated that MA-mediated neuroprotection in the rat embolic middle cerebral artery occlusion model of stroke is dependent on the activation of PI3K/AKT signaling [12]. It has also been reported that increased IL-6 levels within the brain parenchyma of patients correlates with reduced mortality and improved Glasgow outcome scores following TBI [23]. Thus, our current data further supports the suggestion that an acute, temporally limited increase in IL-6 activity within the CNS may be beneficial after TBI.

### 3.3. Unique MA-Mediated Alterations to Upstream Regulators 

Investigation of upstream regulators by IPA helped to further identify the potential roles of other key cytokines in MA-mediated neuroprotection against TBI. For example, TGFβ1 activity was selectively increased only in the cortex of the TSm group. Although TGFβ1 signaling was equally increased within the hippocampus of all three groups, TGFβR2 activity was increased only in the hippocampus of TM and TSm rats. This is intriguing in light of a recently published study. Using a stab wound model of TBI, Divolis et al. (2019) demonstrated that TGFβ1 signaling in reactive glial cells shifted astrocytes and microglia to the anti-inflammatory and tissue reparative “A2”-like and “M0/M2”-like phenotypes, respectively [24]. Therefore, MA may mediate neuroprotection after TBI, particularly in the hippocampus, by acutely increasing TGFβ1 levels. 

In addition to TGFβ1, upstream regulators enriched by IPA also suggested an increase in IL1β activity, but only within the hippocampus of TSm rats. In contrast, we previously reported, based on gene array analysis confirmed by rtPCR, that IL1β gene expression was increased by 4.6-fold (*p* = 0.005) under the same experimental conditions and time point in TS rats but was normalized in TSm rats [13]. Although IL1β is a prototypical pro-inflammatory cytokine in most tissues, it can act as a neuromodulator in healthy brain tissue and may provide protection following injury [25]. Importantly, Song et al. (2013) demonstrated a clear difference between acute and subacute exposure to IL1β within the CNS, with acute exposure inducing neuroprotective effects and subacute exposure promoting neurodegenerative effects. Again, these data suggest that MA may cause the acute activation of IL-1β signaling while limiting its subacute and/or chronic pro-inflammatory activity [26].

Canonical pathways enriched by IPA also revealed that MA treatment uniquely reduced proteins associated with the protein kinase A signaling pathway within the cortex. The activity of the adenosine A2A receptor (ADORA2A), a canonical upstream regulator of the protein kinase A signaling pathway, was also uniquely reduced only within the cortex of TSm rats. Reduction of ADORA2A signaling via pharmacological or genetic approaches significantly improves cognitive outcomes and attenuates cortical and hippocampal lesions, proinflammatory cytokine expression, glutamate release, edema, cell loss, and gliosis in both early and prolonged phases of injury [27,28,29]. Thus, MA may also mediate neuroprotection via acute inactivation of the A2A receptor.

### 3.4. Alterations in Specific Protein Clusters 

Within the network clusters identified as MA-unique, as shown in Figure 5, we observed a total of 5 proteins within the cortex and 14 proteins within the hippocampus that were significantly different between TSm and TS rats. Among these significant changes, the fibrinogen proteins, Fga, Fgb, and Fgg, exhibited the largest fold increase in TSm rats relative to TS controls. This is interesting as TBI is often associated with coagulopathy, which accounts for approximately 30–40% of trauma fatalities [30,31]. Although the mechanisms associated with TBI-induced coagulopathies are poorly understood, a recent study by Samuels et al. (2019) has begun to shed some light on the subject. These authors examined activated clotting time (ACT), angle, maximum amplitude, and functional fibrinogen levels (FFLEV) in 48 TBI patients without polytrauma, compared to 45 TBI patients with polytrauma and 479 polytrauma patients without TBI. Samuels et al. (2019) concluded that isolated TBI was associated with delayed clot formation due to low fibrinogen and that patients may benefit from early replacement of fibrinogen [32]. Thus, the unique increase in Fga, Fgb, and Fgg induced by MA treatment may also serve as a potential neuroprotective mechanism.

Within the same protein cluster (Network 4) as the fibrinogens, we also observed a significant and unique increase in fibronectin (Fn1) levels within the hippocampus of TSm rats only compared to TS controls, as shown in Figure 5. Fibronectin is expressed during tissue repair and exists either as a soluble plasma protein (pFn) or as an insoluble component of the extracellular matrix (cFn). Plasma fibronectin has been shown to play a neuroprotective role following TBI and accumulates within brain tissue following injury [33,34]. Plasma fibronectin-deficient animals perform significantly worse on both motor and cognitive tasks, have significantly increased lesion volume, apoptotic cell death, and had significantly less phagocytic cells in the injured cortex compared to injured mice with normal pFn levels [34]. Recently, Griffiths et al. (2020) reported a significant decrease in fibrinogen levels within the somatosensory barrel fields during the acute phase (15 min–2 h) post TBI [35]. However, a significant increase in fibronectin levels was observed within the hippocampus at three days post injury. The authors suggested that the acute changes in the levels of fibronectin are associated with circuit dismantling, while increases at post-acute time points indicate a potential restorative or regenerative response associated with recovery from TBI [35]. This observation in combination with the cytokine and A2A receptor signaling described above suggest that MA mediates neuroprotection by eliciting broad pleotropic effects. The modulation and timing of these specific signaling pathways relative to TBI and neuroprotection warrant further investigation.

## 4. Materials and Methods 

### 4.1. Animal Experiments

Male Wistar rats (350~400 g) were obtained from Charles River Laboratories (Wilmington, MA, USA) and housed with a 12-h light/dark cycle and ad libitum access to food and water. TBI was induced using the lateral FPI procedure [11]. Briefly, a 5 mm craniotomy was made over the right hemisphere equidistant between the lambda and the bregma and adjacent to the lateral ridge. Animals were given a 20 ms pressure pulse to the intact dura. Mild TBI (TM) rats received a 1.4–1.6 atmospheric pressure pulse. Severe TBI (TS) rats received a 2.6–2.8 atmospheric pressure pulse using an FP302 Fluid Percussion device (AmScien Instruments, Richmond, VA, USA). All animals experiencing apnea were manually ventilated on 0.5-L O2/min until normal breathing occurred. Body temperature, heart rate, and spO2 levels were monitored throughout the surgical procedure. TM rats had an average righting reflex time (time required to become ambulatory) of 5 min. TS rats had an average righting time of 24 min. Neurological Severity Scoring (NSS) was performed by a blinded observer at 6 h post TBI as previously described [13] to define the severity of TBI in individual rats. Scores ranged from 0 to 18, with 18 indicating maximal impairment. Animals with an NSS of <5 or >10 were classified as TM or TS, respectively. TS rats were randomly divided into two groups. The TS group received continuous IV infusion of vehicle (saline) via the femoral vein and the TSm received infusion with MA. Rats receiving a craniectomy only were included as the sham control group (SH). 

TSm rats received an IV bolus of MA at a dose of 0.425 mg/kg, dissolved in sterile saline, followed by continuous IV infusion of MA at 0.5 mg/kg/h for 24 h. SH, TM, and TS rats received a similar infusion with sterile saline only. A total of 39 rats were included in the proteomics study, which encompassed 10 SH, 11 TM, 9 TS, and 9 TSm. Rats were sacrificed 24 h after drug administration began (32 h after TBI) to procure the whole brain. The cortex and hippocampus (ipsilateral to the injury) were separated and snap frozen for sample preparation.

### 4.2. Protein Extraction and Digestion

Dissected rat cortex and hippocampus samples were manually cryopulverized in liquid nitrogen, and 50 mg powdered tissue was weighed and transferred to new Eppendorf tubes for protein extraction. A unique surfactant cocktail-aided extraction/precipitation/on-pellet digestion (SEPOD) protocol was employed to achieve exhaustive and robust protein extraction, as well as efficient and reproducible peptide yields [36]. Briefly, 500 μL surfactant cocktail buffer (containing 50 mM pH 8.4 Tris-formic acid (FA), 150 mM NaCl, 1% sodium deoxycholate, 2% SDS, 2% IGEPAL CA-630), supplemented with cOmplete protease inhibitor tablets (Roche Applied Science, Indianapolis, IN), were added to the weighed tissue. The mixture was homogenized using a Polytron homogenizer (Kinematica AG, Switzerland) by 5~10 times of homogenization-cooling cycles (15,000 rpm) and was subsequently sonicated by 3~5 sonication-cooling cycles (20 s each). The mixture was set on ice for 1 h and then centrifuged at 18,000× *g* under 4 °C for 30 min. Supernatant was transferred to new Eppendorf tubes, and protein concentration of each sample was determined by bicinchoninic acid assay (BCA) assay (Pierce Biotechnology Inc., Rockford, IL, USA).

Based on BCA results, 100 µg of total protein from each sample was aliquoted for proteolytic digestion. Proteins were reduced by 10 mM dithiothreitol (DTT) at 56 °C for 30 min and alkylated by 25 mM iodoacetamide (IAM) at 37 °C for 30 min in darkness. Both reduction and alkylation procedures were performed with rigorous vortexing in an Eppendorf Thermomixer (Eppendorf, Hauppauge, NY, USA). Protein was then precipitated by the addition of 7 volumes of chilled acetone with constant vortexing, and the mixture was incubated under −20 °C for 3 h, followed by 30 min centrifugation at 18,000× *g* under 4 °C to remove supernatant. Pelleted protein was rinsed with 500 µL methanol and left to air dry for 1 min. After wetting the pellets with 80 µL 50 mM Tris-FA, a total volume of 20 µL trypsin (Sigma-Aldrich, St. Louis, MO, USA) dissolved in 50 mM Tris-FA (0.25 µg/µL) was added at an enzyme-to-substrate ratio of 1:20 (*w*/*w*), and tryptic digestion was performed under 37 °C for 6 h with rigorous vortexing in an Eppendorf Thermomixer. Digestion was terminated by addition of 1 µL FA to a final concentration of 1%. Samples were centrifuged at 18,000× *g* under 4 °C for 30 min, and supernatant was carefully transferred to LC vials for analysis.

### 4.3. Nano LC-Orbitrap MS Analysis

For each sample, 4 µL derived peptide (equivalent to 4 µg protein) was separated and analyzed by a nano LC-MS system consisting of a Dionex Ultimate 3000 nano LC system, a Dionex Ultimate 3000 gradient micro LC system with a WPS-3000 autosampler, and an Orbitrap Fusion Lumos mass spectrometer (Thermo Fisher Scientific, San Jose, CA, USA). A large-inner diameter (i.d.) trapping column (300 µm i.d. × 5 mm) was implemented prior to the nano LC column (75 μm i.d. × 100 cm, packed with 3 μm Pepmap C18) for large-capacity sample loading, hydrophilic/hydrophobic matrix component removal, and selective peptide delivery. Mobile Phase A and B were 0.1% FA in 2% acetonitrile and 0.1% FA in 88% acetonitrile. The LC gradient profile was: 4−11% B for 15 min; 11% to 28% B for 110 min; 28% to 44% B for 5 min; 44% to 60% B for 5 min; 60% to 97% B for 1 min, and isocratic at 97% B for 17 min before equilibration to 4% B. MS was operated under data-dependent acquisition (DDA) mode, with a maximal duty cycle time of 3 s. MS1 spectra were acquired in the m/z range 400~1500 under 120 k resolution with default automatic gain control/injection time thresholds and dynamic exclusion settings (60 s ± 10 ppm). Precursor ions were filtered by quadrupole using a 1.2 Th wide window and fragmented by high energy collision dissociation (HCD) at a normalized collision energy of 35%. MS2 spectra were acquired under 15k resolution in Orbitrap. More detailed information about the LC-MS system can be found in Shen et al. [37].

### 4.4. Protein Identification and Quantification

LC-MS raw files were matched to rat SwissProt+TrEMBL database (ver.201608, 35,953 entries) using the MS-GF+ searching engine (v10089, released on 16 July 2013). The search parameters set were listed as follows: (1) Precursor ion mass tolerance: 20 ppm; (2) Instrument type: Q-Exactive; (3) Matches per spectrum: 1; (4) Fixed modification: carbamidomethylation of cysteine; (5) Dynamic modification: oxidation of methionine and acetylation of peptide N-terminal; (6) Maximal missed cleavages per peptide: 2. peptide-spectrum match (PSM) filtering, protein inference/grouping, and global false discovery rate (FDR) control was accomplished by IDPicker (v3.1.18192.0). Both protein and peptide FDR were controlled at ≤1%, while a minimum of 2 unique peptides per protein was required. Proteins with no unique peptides are grouped into 1 protein group with a maximal number of 50 proteins per group. The filtered PSM list was exported and integrated by a customized R script IonStarSPG.R (https://github.com/JunQu-Lab/UHR-IonStar/releases, accessed on 15 January 2021). 

For protein quantification, an in-house developed IonStar MS1-based label-free method was employed to achieve high-precision, low-missing-data quantification in large sample cohorts [15], which involves SIEVE v2.2 (Thermo Fisher Scientific, San Jose, CA, USA) and an in-house developed R-Shiny app UHR-IonStar v1.4 (https://github.com/JunQu-Lab/UHR-IonStar/releases, accessed on 15 January 2021). The major procedures include: (i) Chromatographic alignment with ChromAlign [38] for inter-run retention time (RT) calibration and peak clustering. Quality control and selection of the optimal reference for alignment were accomplished by monitoring alignment scores and base-peak ion chromatogram intensity. (ii) Data-independent MS1 quantitative feature generation using the Direct Ion-Current Extraction (DICE) method, which extracts ion chromatograms for all precursor ions with corresponding MS2 scans in the aligned dataset with a defined m/z-RT window (10 ppm, 1 min). (iii) Integration of the SQL database exported from SIEVE (containing corresponding intensities of all quantitative features in the sample set) and the filtered PSM list by MS2 scan number. Frames with valid PSMs were then subjected to dataset-wide normalization, multivariate outlier detection/removal, and aggregation to protein level using UHR-IonStar. Detailed steps can be found in the user manual (available at https://github.com/JunQu-Lab/UHR-IonStar, accessed on 15 January 2021).

### 4.5. Data Analysis and Bioinformatics

Generated quantitative results were processed by UHR-IonStar for data formatting and cleanup, inter-group ratio calculation, and statistical testing by Student’s t-test. Optimal cutoffs for determining significantly changed proteins in each case-control pair were identified by an experimental null approach described previously [16]. Hierarchical clustering was performed by corresponding R packages. Gene Ontology (GO) analysis was performed by the Database for Annotation, Visualization, and Integrated Discovery (DAVID) Bioinformatics Resources v6.8 (http://david.abcc.ncifcrf.gov, accessed on 15 January 2021) [39]. Biological processes assigned by DAVID were manually curated and filtered. Pathway analysis was performed by Ingenuity Pathway Analysis (https://www.qiagenbioinformatics.com/products/ingenuity-pathway-analysis/, accessed on 15 January 2021) [40] and results were manually exported and compiled. Protein network analysis was performed by STRINGDB, and further processed and visualized by Cytoscape. All data visualization was done by ggplot2 package in R and Graphpad Prism.

## 5. Conclusions

In conclusion, a large-scale quantitative proteomics investigation of molecular mechanisms underlying the neuroprotective effects of MA was conducted in cortical and hippocampal tissues procured from a rat model of TBI. The use of IonStar allowed high-reproducibility, low-missing-data quantification of >7000 proteins in 78 rat brain samples, and two different categories of significant protein (i.e., APs) changes resulting from MA treatment were identified with high confidence. While the MA-induced APs represent “effectual” changes that alleviate neuropathology by preventing the occurrence of “severe TBI-like” neuroproteomic patterns, the MA-unique changes represent “causal” changes that activate specific signaling pathways to elicit neuroprotective effects. Further data analysis and interpretation suggested the involvement of acute activation of TGFβ1, IL1β, and ADORA2A signaling, as well as the increased levels of fibrinogens and fibronectin to block coagulopathies.

## Data Availability

The mass spectrometry proteomics data have been deposited to the ProteomeXchange Consortium via the PRIDE [41] partner repository with the dataset identifier PXD024317 and 10.6019/PXD024317. UHR-IonStar and the user manual is available at (https://github.com/JunQu-Lab/UHR-IonStar, accessed on 15 January 2021).

## Supplementary Materials

Supplementary materials can be found at https://www.mdpi.com/1422-0067/22/5/2246/s1.

## Abbreviations

AP	Altered proteins
BCA	Bicinchoninic acid assay
BP	Biological processes
DTT	Dithiothreitol
EN	Experimental Null
FA	Formic acid
FADR	False Altered-protein Discovery Rate
FC	Fold change
FPI	Fluid percussion injury
GO	Gene Ontology
IAM	Iodoacetamide
IPA	Ingenuity Pathway Analysis
LC	Liquid chromatography
MA	Methamphetamine
MS	Mass spectrometry
NSS	Neurological Severity Score
PCA	Principal Component Analysis
PPI	Protein–protein interaction
ROS	Reactive oxygen species
SEPOD	Surfactant cocktail-aided Extraction/Precipitation/On-pellet Digestion
SH	Sham control
TBI	Traumatic brain injury
TM	Mild TBI
TS	Severe TBI
TSm	Severe TBI with MA treatment
UHF	Ultra-high-field
