# Supplementary material for: Potential Neuroprotective Mechanisms of Methamphetamine Treatment in Traumatic Brain Injury Defined by Large-Scale IonStar-Based Quantitative Proteomics
Source: Int J Mol Sci. 2021 Feb 24;22(5):2246. doi: 10.3390/ijms22052246 (PMC7956755; doi:10.3390/ijms22052246)
Supplement: Supplementary file 1 [file ijms-22-02246-s001.zip › ijms-1106736 (supp)-for publication.pdf]

Potential Neuroprotective Mechanisms of Methamphetamine Treatment in Traumatic Brain Injury Defined by Large-Scale IonStar-Based Quantitative Proteomics

Shichen Shen [1,2], Ming Zhang [1,2], Min Ma [2,3], Sailee Rasam [2,4], David Poulsen [5] and Jun Qu [1,2,*]

1 Department of Pharmaceutical Sciences, University at Buffalo, Buffalo, NY 14214, USA; ShichenS@buffalo.edu (S.S); MZhang25@buffalo.edu (M.Z); JunQu@buffalo.edu (J.Q)

2 New York State Center of Excellence in Bioinformatics and Life Sciences, Buffalo, NY 14203, USA

3 Department of Cell Stress Biology, Roswell Park Comprehensive Cancer Institute, Buffalo, NY 14203, USA; MinMa@buffalo.edu (M.M)

4 Department of Biochemistry, Jacobs School of Medicine and Biomedical Sciences, Buffalo, NY 14203, USA; SaileeSu@buffalo.edu (S.S)

5 Department of Neurosurgery, Jacobs School of Medicine and Biomedical Sciences, Buffalo, NY 14203, USA; DavidPou@buffalo.edu (D.P)

Supplementary Materials

Supplementary Figure S1 – S5.

Supplementary Table S1 – S4.

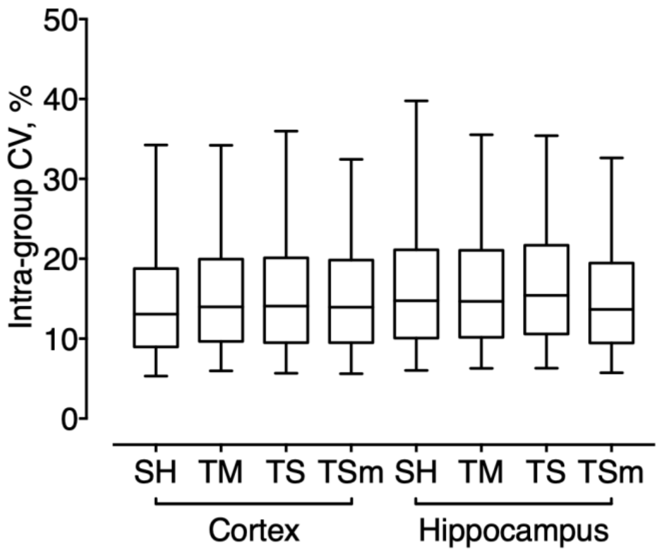

Supplementary Figure S1. Intra-group coefficient of variation (CV) of biological replicates from cortex and hippocampus.

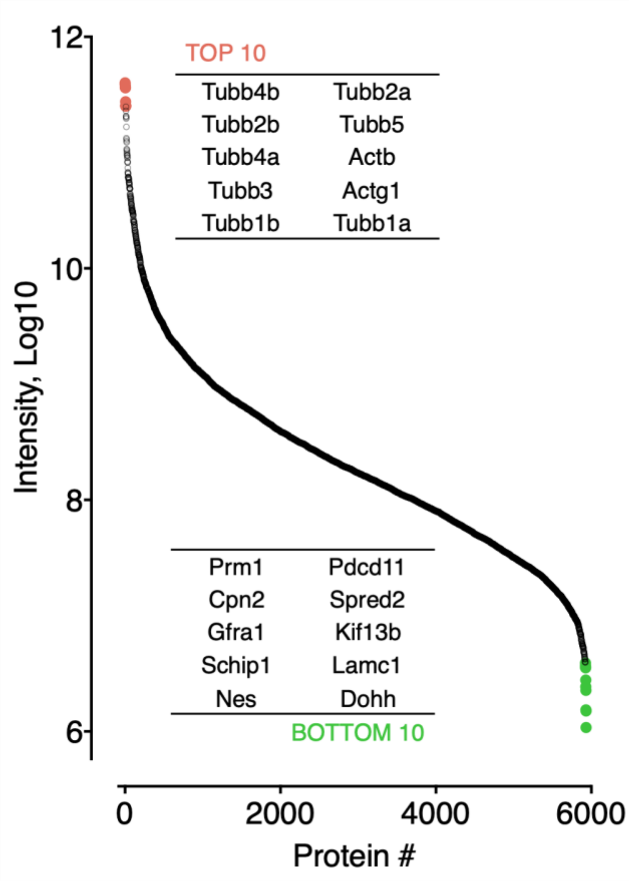

Supplementary Figure S2. Abundance rank of proteins quantified in the study. Proteins with the highest (TOP10) and lowest (BOTTOM10) abundances are highlighted.

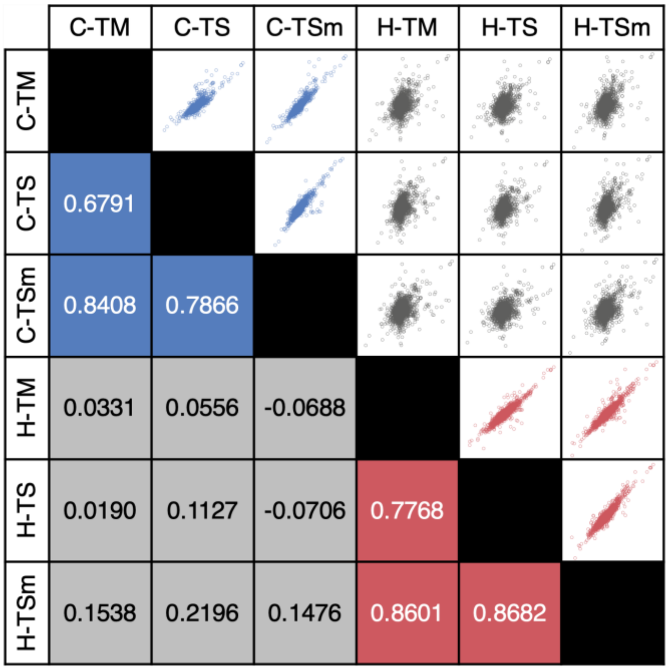

Supplementary Figure S3. R-squared values from Pearson correlation of protein ratios (compared to the SH group).

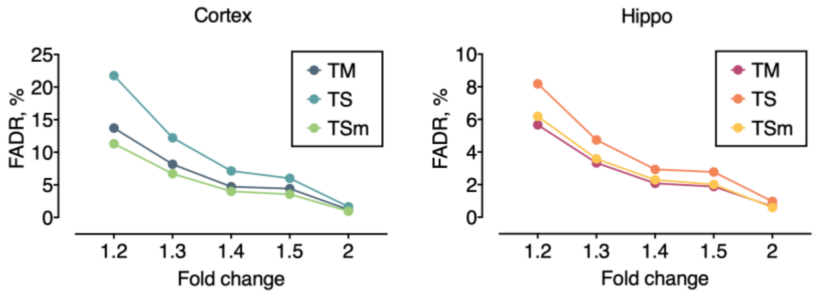

Protein #

Group	Cortex					Hippocampus				
	1.2-fold	1.3-fold	1.4-fold	1.5-fold	2-fold	1.2-fold	1.3-fold	1.4-fold	1.5-fold	2-fold
TM	685	404	297	204	83	1660	991	674	479	152
TS	432	270	196	150	61	1148	696	477	324	103
TSM	831	492	350	253	104	1520	922	611	447	168

FADR, %

Group	Cortex					Hippocampus				
	1.2-fold	1.3-fold	1.4-fold	1.5-fold	2-fold	1.2-fold	1.3-fold	1.4-fold	1.5-fold	2-fold
TM	13.7	8.2	4.7	4.4	1.2	5.7	3.3	2.1	1.9	0.7
TS	21.8	12.2	7.1	6.0	1.6	8.2	4.8	2.9	2.8	1.0
TSM	11.3	6.7	4.0	3.6	1.0	6.2	3.6	2.3	2.0	0.6

Supplementary Figure S4. Optimization of protein fold change (FC) thresholds for determining altered proteins (APs) using an Experimental Null (EN) method. 1.4-fold protein FC was selected based on the consideration to control False Altered-protein Discovery Rate (FADR) low and obtain a reasonable number of APs.

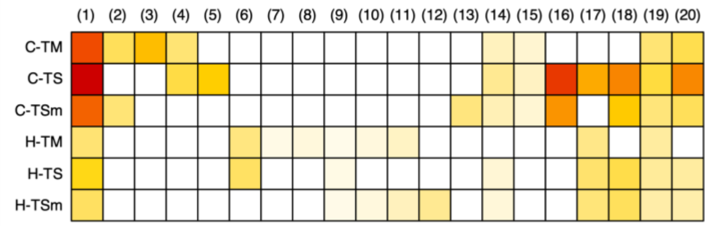

#	GOBP Terms	#	GOBP Terms
1	Oxygen transport	11	Positive regulation of cell death
2	Vasodilation	12	Response to amine
3	Chronic inflammatory response	13	Complement activation, classical pathways
4	Retina homeostasis	14	Negative regulation of endopeptidase activity
5	Cellular response to IL-6	15	Innate immune response
6	Astrocyte development	16	Protein polymerization
7	Translation	17	Hydrogen peroxide catabolic process
8	Actin cytoskeleton organization	18	Fibrinolysis
9	Negative regulation of apoptotic process	19	Platelet aggregation
10	Cell-cell adhesion	20	Acute-phase response

Supplementary Figure S5. Gene Ontology Biological Processes (GOBP) enriched from APs using DAVID.

Supplementary Table S1. Quantification results of rat cortical tissues.

Supplementary Table S2. Quantification results of rat hippocampal tissues.

Supplementary Table S3. Altered proteins (APs) of rat cortical tissues and classification results.

Supplementary Table S4. Altered proteins (APs) of rat hippocampal tissues and classification results.

**All Supplementary Tables are provided as separate Excel files.
